# Coronary artery surgery in a man with achondroplasia: a case report

**DOI:** 10.1186/1752-1947-4-348

**Published:** 2010-10-29

**Authors:** Georgios I Tagarakis, Dimos Karangelis, Andony J Baddour, Nicholas Desimonas, Angeliki Tsantsaridou, Marios E Daskalopoulos, Dimitrios Papadopoulos, Nikolaos B Tsilimingas

**Affiliations:** 1 Department of Cardiovascular and Thoracic Surgery, University Hospital of Thessaly, Larissa, Greece

## Abstract

**Introduction:**

Achondroplasia is a musculoskeletal disorder associated with short stature. Despite an estimated prevalence of 1:25,000 in the general population, there is little literature concerning the diagnostic and treatment challenges faced by doctors dealing with a heart operation on a patient with this condition.

**Case presentation:**

We present the case of a 41-year-old Caucasian man of Greek ethnicity with achondroplasia, who underwent bypass heart surgery.

**Conclusions:**

The surgery was successful and did not present particular difficulties, showing that heart surgery can be safely performed on people with achondroplasia.

## Introduction

Dwarfism is a term referring to people of short stature, and can be due to several musculoskeletal and hormonal growth disorders. The most common cause is considered to be achondroplasia, a morbid condition due to a mutation affecting the fibroblast growth factor receptor (FGFR) gene 3. Despite an estimated prevalence of 1:25,000 in the general population [1], little has been written on the diagnostic and treatment challenges faced by doctors with achondroplasia patients who require heart surgery. We report here a third case concerning dwarfs and heart surgery and the second dealing with dwarfs and bypass heart surgery [2,3].

## Case presentation

A 41-year-old Caucasian man of Greek ethnicity with achondroplasia was referred to our cardiothoracic surgery department following coronary artery angiography and a diagnosis of three-vessel coronary artery disease. Angiography was performed due to mild anginal symptoms dating about four months prior to the examination. Interestingly, our patient lacked any predisposing risk factors, including burdening family history, with the exception of a borderline arterial blood pressure of 135 to 140/85 to 90 mmHg. His lipidological/biochemical profile included the following values: total serum cholesterol 190 mg/dL, LDL (low density lipoprotein) cholesterol 148 mg/dL, HDL (high density lipoprotein) cholesterol 45 mg/dL, creatinine phosphokinase 100 U/L, creatinine phosphokinase-MB 7 U/L. Due to the short stature of our patient (137 cm height, 45 kg weight) and the disproportionate skeletal growth (short limbs, normal size thorax, big head), we performed a phlebography of the limbs to check the status of possible saphenous vein grafts, which proved to be normal. Our patient was also submitted to routine pre-operative control and scheduled for an elective on pump coronary artery bypass graft (CABG) operation. The official indication for the surgical revascularization of our patient was based to his three-vessel disease, which included a 90% stenosis of the proximal left anterior descending artery, a 70% stenosis of the proximal obtuse marginal artery and a 90% stenosis of the right coronary artery. Both echogardiography and ventriculography revealed no additional pathologies, with a left ventricular ejection fraction of 65%.

At surgery, anatomical conditions in the thorax appeared normal and a triple on-pump bypass operation with one arterial (left internal mammary artery to left anterior descending artery) and two venous (vena saphena magna) grafts (to the first obtuse marginal branch and the right coronary artery before the crux) was performed without any problems, with anesthetic and cardioplegic adjustments (St. Thomas cardioplegic solution at 32°C) made only to fit the patient's body mass index and weight. The patient had a flawless postoperative course and was discharged from the clinic on the tenth post-operative day.

Follow-up controls one week, one month and six months after discharge revealed no pathologies and our patient was objectively and subjectively well.

## Discussion

A first issue to be addressed is the fact that a young patient with achondroplasia developed operation-indicated coronary artery disease without detectable risk factors. Does this maybe mean that occult genetic or other factors in patients with dwarfism might lead to vascular morbid situations, such as coronary artery disease, or was this a random event? The matter warrants further investigation, as our patient is, to the best of our knowledge, only the second in the medical literature, and as a result little experience has been gathered.

The second issue is that dwarfs with achondroplasia can safely undergo heart surgery with analogical adjustments made accordingly to the patient's body mass index and weight.

A third issue is the small number of case reports dealing with this subject. Are people with dwarfism less frequently submitted to heart surgery than the general population? If this is the case, are there any medical reasons for that or must we search for other causes, such as the fear of doctors to deal with such patients due to lack of experience or the social exclusion of dwarfs in some societies, for example.

## Conclusions

Heart surgery can be safely performed on patients with dwarfism due to achondroplasia, despite the fact that little information dealing with this issue is available in international literature. It is interesting that only a small number of similar reports have been published.

## Competing interests

The authors declare that they have no competing interests.

## Consent

Written informed consent was obtained from the patient for publication of this case report and any accompanying images. A copy of the written consent is available for review by the Editor-in-Chief of this journal.

## Authors' contributions

GT was the primary surgeon of this case, made a thorough literature review, and was the chief author in terms of developing the paper. DK performed a consultation regarding anti-reflux treatment, and co-authored the paper. AB was the attending surgeon of the case and checked the paper. ND assisted with the linguistics and performed a literature research. AT performed literature research. MD checked the final version of the manuscript. PD assisted with the literature research. NT was the attending surgeon. All authors read and approved the final manuscript.
